# Draft Genome Sequence of *Erwinia billingiae* OSU19-1, Isolated from a Pear Tree Canker

**DOI:** 10.1128/genomeA.01119-15

**Published:** 2015-10-01

**Authors:** Jeannie M. Klein, Rhett W. Bennett, Logan MacFarland, Megan E. Abranches Da Silva, Britney M. Meza-Turner, Phillip M. Dark, Mackenzie E. Frey, Dulani P. Wellappili, Aron D. Beugli, Holman J. Jue, Joshua M. Mellander, Wei Wei, Walt Ream

**Affiliations:** Department of Microbiology, Oregon State University, Corvallis, Oregon, USA

## Abstract

Plant-associated *Erwinia* include pathogenic and nonpathogenic species*.* We report the 5.6-Mb genome sequence of *Erwinia billingiae* OSU19-1, isolated from a canker on a pear tree inoculated with *Erwinia amylovora*. OSU19-1 and a closely related European isolate, *E. billingiae* Eb661^T^, share many similarities including 40 kb of plasmid sequence.

## GENOME ANNOUNCEMENT

Plant-associated Gram-negative *Erwinia* spp. belong to the *Enterobacteriaceae* family. Species include plant epiphyte *Erwinia billingiae* and pathogenic *Erwinia amylovora*, which causes fire blight (1, 2). We isolated *E. billingiae* OSU19-1 from a canker on the mid-trunk cambium of a Concorde pear tree inoculated with *E. amylovora* 1 year prior; others also found *E. billingiae* in necrotic tissue of trees with fire blight (1–3). We surface sterilized tissue in 10% bleach, rinsed with sterile water, pulverized tissue in sterile water, and isolated bacteria on LB agar at 25°C. We used a MoBio PowerSoil kit to purify genomic DNA from bacteria cultured in LB broth (4).

To compare *E. billingiae* OSU19-1 with Eb661^T^, the type strain isolated in England in 1959, we sequenced the genome of OSU19-1 using an Illumina MiSeq to generate 250-bp paired-end reads. Low-quality read pairs flagged by the MiSeq were removed prior to quality trimming with Sickle (quality score = 30; minimum length = 50), yielding 2,172,379 read pairs; single reads were discarded. To obtain optimum coverage (~65×), we used 663,000 read pairs to complete four *de novo* assemblies with ABySS, Celera Assembler, IDBA, and Velvet (5–9). Optimum k-mer lengths of 65, 120, and 87 were used for the ABySS, IDBA, and Velvet assemblies, respectively. We used Minimus2 to merge the Celera and Velvet assemblies; ABySS and IDBA assemblies were merged similarly (10). These combined assemblies were merged into a consensus alignment, which was validated using REAPR (11). The final assembly contained 5,602,087 bp (55% G/C) in 32 contigs (*N*_50_, 409,442 bp; maximum, 668,909).

Annotation using RAST (12) predicted 4,931 protein coding sequences and 76 RNAs. BLASTn analysis of *recA*, *gyrA*, *gyrB*, and *gpd* genes revealed 98 to 99% identity between OSU19-1 and its closest known relative, *E. billingiae* Eb661^T^ (3); small-subunit rRNA genes were identical. OSU19-1 encodes several quorum-sensing systems, including LuxS, which produces autoinducer-2, and two homoserine lactone synthases. Similarly, Eb661^T^ produces an acyl-homoserine lactone (13) and autoinducer-2 (14). OSU19-1 and Eb661 lack a type III secretion system, which is important for pathogenicity of *E. amylovora* (3).

The 5.37-Mb genome of Eb661^T^ has a chromosome of 5,100,168 bp and two plasmids, pEB102 (102 kb) and pEB170 (170 kb) (3). OSU19-1 lacks significant similarity to pEB170, but contigs 2 (98,580 bp) and 10 (40,687 bp) share 26,170 bp and 14,490 bp (94 to 96% identity) with different regions of pEB102. The portion of contig 2 homologous to pEB102 includes four genes resembling those in the integrative conjugative element Genomic Island-1 of *Pseudomonas fluorescens* Pf-5 (15). Outside this shared region, contig 2 encodes RepA and ParAB proteins; it also encodes DNA repair proteins UmuC and RadC, which may help *E. billingiae* survive exposure to UV radiation. Study of genetic diversity in *E. billingiae* increases understanding of this microbe’s lifestyle and its potential for biocontrol of fire blight (3, 13).

### Nucleotide sequence accession numbers. 

This whole-genome shotgun project has been deposited at DDBJ/EMBL/GenBank under the accession no. LHXI00000000. The version described in this paper is version LHXI01000000.
